# Prognostic significance of magnetic resonance imaging parameters in patients with idiopathic dilated cardiomyopathy

**DOI:** 10.1186/1532-429X-11-S1-O17

**Published:** 2009-01-28

**Authors:** Nico Merkle, Jan Torzewski, Georg Grossmann, Volker Rasche, Matthias Kochs, Jochen Woehrle, Vinzenz Hombach

**Affiliations:** University Ulm, Ulm, Germany

**Keywords:** Ejection Fraction, Left Ventricular Ejection Fraction, Cardiac Death, Endsystolic Volume, Prognostic Parameter

## Background

Patients with idiopathic dilated cardiomyopathy (IDC) have a limited prognosis. Aim of this study was to evaluate the prognostic significance of novel magnetic resonance imaging (CMR) parameters in IDC patients.

## Methods and results

161 patients with IDC were studied by CMR for hemodynamic and late enhancement (LE) analysis and followed for a mean of 933,8 ± 529,2 days. QRS and QTc intervals were measured from 12-lead ECGs. LV and RV enddiastolic and endsystolic volume indexes were increased and ejection fractions (EF) decreased (LV-EF 33 ± 14%, RV-EF 50 ± 16%). LE was seen in 43 patients (27%). Interventricular dyssynchrony (50.9 ± 67.2 ms) was present in 68 patients (42%). Primary endpoint was cardiac death, sudden death (SCD) and rehospitalization for pump failure, secondary endpoint cardiac death and SCD. 3 patients died from non-cardiac, 10 patients from cardiac death, 2 patients from SCD and additional 3 patients had ICD shock for ventricular flutter/fibrillation, and 35 patients were rehospitalized. Multivariate analysis revealed depressed left ventricular EF(<25%) right ventricular EF (<30%), as well as the presence of LE as independent prognostic parameters. Kaplan-Meier survival analysis displayed low LV (<25%) and RV (<30%) ejection fraction and the presence of LE as significant parameters for a worse outcome.

## Conclusion

In addition to impaired left ventricular ejection fraction (<25–30%) a depressed right ventricular function (EF <30%) and the presence of late enhancement derived from CMR are novel prognostic parameters in patients with IDCM.

